# Role and Mechanism of LIF in Oral Squamous Cell Carcinoma Progression

**DOI:** 10.3390/jcm9020295

**Published:** 2020-01-21

**Authors:** Ting-An Lin, Tai-Sheng Wu, Yue-Ju Li, Cheng-Ning Yang, Monica Maria Illescas Ralda, Hao-Hueng Chang

**Affiliations:** 1 Graduate Institute of Oral Biology, School of Dentistry, National Taiwan University, Taipei 100, Taiwan; ting4357025@gmail.com; 2 Graduate Institute of Clinical Dentistry, School of Dentistry, National Taiwan University, Taipei 100, Taiwan; eric12345a@hotmail.com (T.-S.W.); anaballlee@yahoo.com.tw (Y.-J.L.); cnyang880@yahoo.com.tw (C.-N.Y.); mmillescas@ufm.edu (M.M.I.R.); 3 Department of Surgery, National Taiwan University Hospital, Taipei 100, Taiwan; 4 Department of Dentistry, National Taiwan University Hospital, Taipei 100, Taiwan

**Keywords:** oral cancer, LIF, INHBA, migration, invasion

## Abstract

Background: Metastasis is a severe problem in patients with oral squamous cell carcinoma (OSCC), which is the fifth most common cancer worldwide. Leukemia inhibitory factor (LIF) has been studied in different cancers, while the role of LIF in OSCC remains unclear. Methods: LIF expression was detected in 100 OSCC samples by immunohistochemistry. Effects of LIF on cell motility were evaluated in OSCC cell lines. High-throughput microarray analysis was also conducted. The correlation between LIF and the downstream effector was analyzed by real-time quantitative reverse transcription PCR. Results: Patients with OSCC who had lymph node metastasis or advanced cancer stages showed high LIF expression. OSCC patients with higher LIF expression, advanced stage, large tumor size, or lymph node metastasis had significantly shorter overall survival. LIF regulated cancer cell motilities through outside-in signaling. The *inhibin beta A subunit* (*INHBA*) gene was identified as a crucial downstream effector of LIF-promoted OSCC progression and restored migration and invasion abilities in LIF knockdown transfectants. Conclusion: LIF enhances regional lymphatic spread, thus leading to an advanced cancer stage. Regulation of LIF downstream molecules such as INHBA inhibits the invasion or migration ability of cancer cells. Thus, LIF can be a potential target in preventing cancer metastasis and spread.

## 1. Introduction

Oral cancer is the sixth leading cause of malignant tumor mortality and is an important healthcare problem worldwide [1]. Oral squamous cell carcinoma (OSCC) accounts for more than 90% of all oral cancers, with over 300,000 new cases being registered annually [2,3]. Oral cancer is an umbrella term for cancers that originate in sites such as the tongue, lip, gums, buccal mucosa, floor of the mouth, oropharynx, gingiva, and hard palate [2,4]. The risk factors for oral malignancy are tobacco smoking, alcoholism, an unhealthy diet, and viral infections [2,4]. Unlike in Western countries, in Southeast Asia and Taiwan, extensive epidemiologic evidence has demonstrated a close relationship between the development of OSCC and areca nut chewing, tobacco smoking, and alcohol consumption [5,6,7]. According to a report by the Ministry of Health and Welfare, Taiwan, oral cancer has the seventh highest incidence and the sixth highest mortality rate of all cancers [8,9].

In Taiwan, those who chew betel nuts have a higher relative risk of oral cancer than those who do not. Arecoline, the main component of areca nut extract, is a major risk factor for oral premalignant lesions and considerably increases OSCC-related carcinogenesis [10]. In addition, areca nut components stimulate epidermal growth factor receptor (EGFR) phosphorylation and interleukin (IL)-1α production and induce reactive oxygen species in oral submucous fibrosis [11]. Tobacco smoking and alcohol consumption are also key risk factors for oral cancer in Taiwan. Smokers have a higher risk of potentially malignant oral disorders (hazard ratio of 2.8) than nonsmokers [12]. *ADH1B*, one of the genes encoding the ADH enzyme, and *ALDH2*, a gene that encodes an enzyme responsible for the conversion of acetaldehyde to acetate, may be associated with head and neck cancer in alcohol drinkers [13].

Leukemia inhibitory factor (LIF) is a pleiotropic proinflammatory cytokine in the IL-6 group. LIF is widely expressed in different types of tissues and regulates multiple vital biological functions such as survival, differentiation, and inflammatory response [14,15]. It is so named because of its function of inducing the differentiation of murine M1 myeloid leukemia cells and macrophage maturation to suppress leukemia proliferation [16]. LIF binds to the LIF receptor (LIFR) and glycoprotein (gp130, IL6ST) to form a high-affinity receptor complex. This heterodimer then activates many downstream signaling pathways of LIF, including JAK/STAT3, PI3K/AKT, MAPK/ERK, and mTOR, and regulates transcription [17,18,19,20].

LIF has been reported to have distinct effects on tumorigenesis and metastatic spread in different cancers. It exerts tumor-suppressing effects through TGF-β-induced cell cycle arrest and inhibition of cell migration through the LIF/p21 signaling cascade pathway in cutaneous melanoma [21]. Chen et al. found that LIFR suppresses breast cancer metastasis by triggering the Hippo–YAP pathway to inhibit local invasion and metastatic colonization [22,23]. LIF is also considered to be an oncogene that promotes the development and progression of many types of solid tumors. LIF and LIFR are overexpressed in nasopharyngeal carcinoma and were shown to enhance radioresistance through activating mTOR and p70S6K [18]. In rhabdomyosarcoma, LIF stimulates AKT phosphorylation and strongly enhances the invasive potential of tumors [24]. Furthermore, LIF overexpression can promote colorectal cancer chemoresistance in a p53-dependent manner [25].

Although LIF has been indicated as a novel biomarker and a prognostic target in various human cancers, its biological functions in oral cancer remain unknown. Also, the relationship between LIF and the risk factors of oral cancer such as tobacco smoking, betel nut chewing, and alcohol consumption is unclear. In this study, we evaluated the correlation between LIF expression and the clinicopathological parameters of patients with OSCC and investigated whether LIF drives tumor progression in oral cancer.

## 2. Materials and Methods

### 2.1. Cell Lines and Culture

SAS (JCRB 0260), CA9-22 (JCRB 0625), and Cal27 (ATCC CRL-2095) purchased from ATCC were used in this study. All cell lines were cultured in Dulbecco’s modified Eagle’s medium (Gibco, Grand Island, NY, USA) supplemented with 10% fetal bovine serum (Gibco) and 1% antibiotics (Gibco) at 37 °C in a humidified atmosphere of 5% CO_2_ and 95% air. For routine culture and harvesting, adherent cells were trypsinized from culture dishes with 0.05% trypsin/EDTA (Gibco).

### 2.2. Transient Transfection and Stable Transfection Clone Selection

shLIF plasmids (Academia Sinica RNAi Core Lab, Taipei, Taiwan), LIF (Origene, Rockville, MD, USA, NM_002309), and *inhibin beta A subunit* (*INHBA*) (Origene, NM_002192) expression plasmids were transiently transfected into OSCC cell lines by Lipofectamine 2000 (Invitrogen, Carlsbad, CA, USA), and the transfection protocol was conducted according to the manufacturer’s instructions (Invitrogen, Carlsbad, CA, USA). Integration of transfectant plasmid DNA was confirmed by Western blotting. After transection for 24 h, the stable transfectant was selected in puromycin at a concentration of 3 µg/mL.

### 2.3. Western Blotting

Cells were collected with a lysis buffer. Proteins (50 µg) were separated by sodium-dodecyl-sulfate–polyacrylamide-gel-electrophoresis on 10% gels and electrotransferred to a polyvinylidene difluoride (PVDF) membrane (Millipore, Bedford, MA, USA). After the blot was blocked in 5% skim milk with 0.1% Tween-20, membrane-bound proteins were probed with primary antibodies at 4 °C overnight and incubated with peroxidase-conjugated polyclonal secondary antibodies (1:3000) for 1 h at room temperature. Antibody-bound protein bands were detected with enhanced chemiluminescence reagents (Millipore) and photographed with an LAS-4000 (Fujifilm, Tokyo, Japan). LIF (Abcam, Cambridge, UK, ab135629), INHBA (R & D systems, Minneapolis, MN USA, AF338), and β-actin (Santa Cruz Biotechnology, Dallas, TX, USA, sc-47778) antibodies were used for the Western blotting analysis.

### 2.4. Immunohistochemistry

Immunohistochemical studies were conducted using the ss-polymer HRP detection kit (Biogenex, San Ramon, CA, USA). Briefly, after being deparaffinized and rehydrated, the sections were subjected to 10 min of heat-induced antigen retrieval in citrate buffer (pH 6.0). After being blocked by 3% hydrogen peroxide, the sections (slides) were incubated with primary antibodies (monoclonal anti-LIF (1:100)) at 4 °C overnight. Then, the slides were incubated for 60 min with super enhancer and 20 min with the polymer HRP. Antigen–antibody complexes were visualized with 3.3 hematoxylin, differentiated, dehydrated, and mounted. A routinely processed OSCC that was previously proven to be positive for LIF was used as a positive control, and the OSCC section without a primary antibody served as a negative control in each staining series. The grade of LIF protein expression in each specimen was further classified by intensity level, namely, 0 (negative or <5% of tumor cells stained), 1 (5–25% of tumor cells stained), 2 (26–50% of tumor cells stained), 3 (51–75% of tumor cells stained), and 4 (>76% of tumor cells stained). Two authors (H.-H.C. and M.M.I.R.) reviewed the LIF staining on each slide independently without knowledge of the patients’ information.

### 2.5. In Vitro Migration and Invasion Assay

For in vitro cell invasion and migration assays, Transwell chambers (8 μm pore size; Corning Costar, Cambridge, MA, USA) coated with or without Matrigel (Corning, New York, NY, USA) in 24-well dishes were used. Cells were allowed to grow to subconfluency (~80–90%). After detachment with trypsin, cells resuspended in a serum-free medium and 100 µL cell suspension (1 × 10^5^ cells/mL) were added to the upper chamber. A complete medium was added to the bottom wells of the chambers. After 24 h, the cells were fixed in ice methanol for 15 min and the upper side cells of the filters were removed with cotton-tipped swabs. The filters were then cleared with phosphate-buffered saline (PBS) and the cells were stained with 0.05% crystal violet in PBS for 20 min. The underside cells of the filters were viewed, images of 10 different fields were captured from each membrane, and the number of migratory cells was counted. The mean of triplicate assays for each experimental condition was used.

### 2.6. RNA Isolation

RNA was isolated from oral carcinoma cells and samples from patients with OSCC by TRIzol (Invitrogen, Carlsbad, CA, USA). Reverse transcription was performed in a final reaction containing the following: total RNA (5 µg), First Strand Buffer with DTT (10 mM), deoxyribonucleotide triphosphate (dNTP; 2.5 mM), Oligo (dT) 12–18 primer (1 µg), and Moloney murine leukemia virus reverse transcriptase (200 U). The reaction was incubated at 65 °C for 5 min and then was terminated by heating at 42 °C for 1 h.

### 2.7. Reverse Transcription PCR

PCR amplification was conducted in a reaction buffer containing 20 mM of Tris-HCl (pH 8.4), 50 mM of KCl, 1.5 mM of MgCl_2_, 167 µM of dNTPs, 2.5 U of Taq DNA polymerase, and 0.1 µM of primers. The reactions were performed in a Biometra Thermoblock (Biometra Inc., Baltimore, MD, USA) using the following process: denaturing for 1 min at 95 °C, annealing for 1 min at 58 °C, and elongating for 1 min at 72 °C (30 cycles in total); the final extension occurred at 72 °C for 10 min. Equal volumes of each sample were subjected to electrophoresis on a 1% agarose gel, which was then stained with ethidium bromide and photographed under UV illumination.

### 2.8. Real-Time Quantitative Reverse Transcription PCR

The complementary DNA was used as a template in real-time quantitative PCR reactions with LIF, INHBA, and GAPDH primers using an ECO Sequence detector (Illumina, San Diego, CA, USA) at 95 °C for 10 min, followed by 40 cycles of 95 °C for 15 s and 60 °C for 1 min. Target gene expression was normalized between different samples based on the values of *GAPDH* RNA expression.

### 2.9. OSCC Tumor Samples and Clinical Data Collection

OSCC specimens were collected at the time of surgery from previously untreated patients who underwent surgical resection at National Taiwan University Hospital (201503035RINC). The trial was approved by the Institutional Review Board of National Taiwan University Hospital (registration 201503035RINC). All participants provided informed consent before participating in the trial. We obtained formalin-fixed, paraffin-embedded specimens from 100 patients (91 men and 9 women, mean age of 55.8 years, range of 34–82 years) with OSCC. The diagnosis of OSCC was based on the histological examination of hematoxylin-and-eosin-stained tissue sections. All patients underwent total surgical excision of their OSCCs at the Department of Oral and Maxillofacial Surgery of National Taiwan University Hospital, Taipei, Taiwan. None of the patients had received any form of tumor-specific therapy prior to total surgical excision of their lesions. Specimens were obtained from the total surgical excision of the lesions. Of the 100 cases of OSCC, 48 (48%) were located in the buccal mucosa, 34 (34%) on the tongue, 12 (12%) on the gingiva, 5 (5%) on the hard palate, and 1 (1%) on the floor of the mouth. All of the specimens were snap-frozen immediately and stored at −80 °C. The histologic identification of oral cancer was determined as recommended by the World Health Organization. Tumor size, local depth of invasion (DOI), margin status, and lymph node metastasis were determined on pathologic examination. The final disease stage was determined by a combination of surgical and pathologic findings according to the current tumor–node–metastasis staging system for oral cancer. Follow-up data were obtained from the patients’ medical charts and our tumor registry service.

### 2.10. mRNA Microarray Assay

Total RNA was isolated from cell lines with Trizol (Invitrogen Corporation, Carlsbad, CA, USA). The Human OneArray v5 (Phalanx Biotech Group, Hsinchu, Taiwan) contains 30,275 DNA oligonucleotide probes, and each probe is a 60 mer probe designed in the sense direction. Among the probes, 29,187 probes corresponded to the annotated genes in the Refseq v38 (National Center for Biotechnology Information, Bethesda, MD, USA) and Ensembl v56 (Ensembl, Hinxton, Cambridge, UK) databases.

### 2.11. Statistical Analysis

Data are represented as mean ± SEM. Statistical analyses were performed using an unpaired, two-tailed Student’s *t* test, and the values are expressed as means with 95% confidence intervals. A *p*-value of <0.05 was considered statistically significant. The correlation between the clinicopathological parameters of patients with oral cancer and the expression of LIF was analyzed using the Kruskal–Wallis test. The correlation between LIF expression and habits related to betel nut chewing and tobacco smoking was analyzed using the chi-squared test. The associated prognostic factors were identified by the univariate and multivariate survival analyses with Cox proportional hazards regression model using SAS 9.1 (SAS Institute Inc., Morrisville, NC, USA).

## 3. Results

### 3.1. LIF Affects Regional Lymph Node Involvement and the Advanced Stage of OSCC

**Figure 1:**
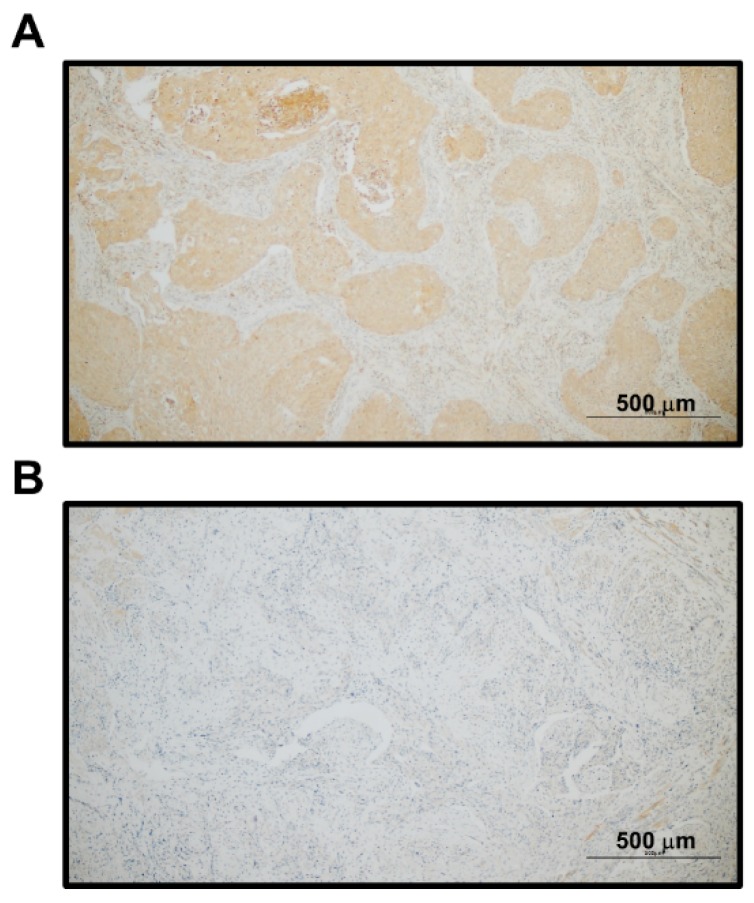
Immunohistochemical staining of LIF expression in patients with OSCC. LIF expression was determined by immunohistochemistry in paraffin-embedded tumor tissues of patients with OSCC. (**A**) Representative image of positive LIF staining in a patient with OSCC. The cytoplasmic LIF staining was found in nearly all cancer cells of the tumor nests. (**B**) Representative image of no LIF staining in a patient with OSCC.

**Figure 2:**
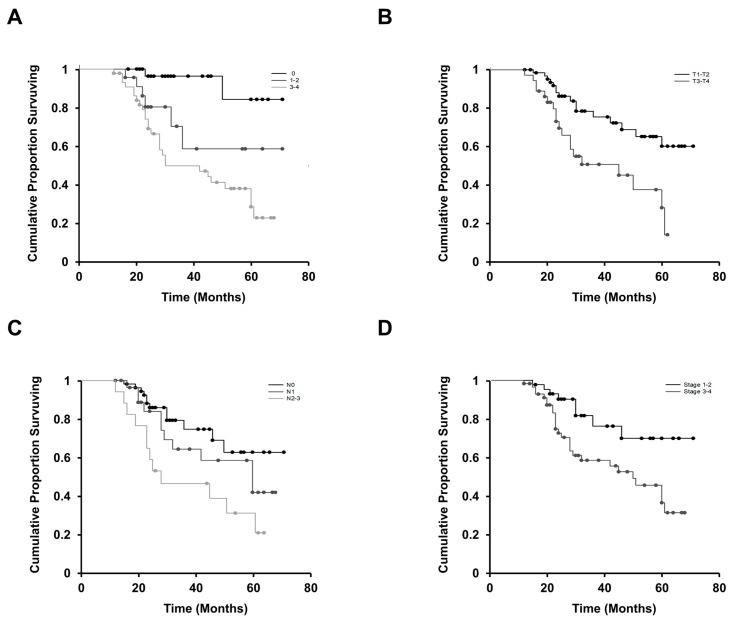
Kaplan–Meier survival curves of 100 patients with OSCC. (**A**) The cumulative survival for patients with none or a low degree (0–2) of LIF staining was significantly higher than that for patients with a high degree (3–4). (**B**) Overall survival was significantly lower in patients with a larger tumor size (T3 + T4) than in those with smaller tumor size (T1 + T2) (*p* = 0.002). (**C**) Overall survival was significantly higher in patients without lymph node metastasis (N0) than in those with an advanced status of lymph node metastasis (N2 + N3) (*p* = 0.014). (**D**) Overall survival was significantly shorter in patients with advanced-stage (stages 3–4) tumors than in those with earlier-stage (stages 1–2) tumors (*p* = 0.011). The duration of survival was measured from the beginning of treatment to the time of death (complete) or the last follow-up (censored).

To study the clinical relevance of LIF in patients with OSCC, we first examined LIF protein expression in paraffin-embedded tumor tissue specimens of patients with OSCC through immunohistochemical analysis. Cytoplasmic LIF staining was found in nearly all cancer cells of the tumor nests for positive LIF staining (Figure 1). The grade of LIF expression in each specimen was further classified based on the percentage of tumor cells stained as follows: level 0 (0% or <5%), level 1 (5–25%), level 2 (26–50%), level 3 (51–75%), and level 4 (>75%). Of the 100 OSCC samples analyzed, negative (level 0), low (levels 1 and 2), and high (levels 3 and 4) expression levels of LIF were observed in 32% (32/100), 22% (22/100), and 46% (46/100) of OSCC specimens, respectively. The relationships between LIF expression in the initial biopsy specimens and clinical parameters of 100 patients with OSCC are shown in Table 1. Cancer with positive lymph node metastasis was associated with higher LIF expression (*p* = 0.022). Similarly, a significant association between LIF staining and advanced cancer staging (stages III and IV) (*p* = 0.002) was noted. Generally, the larger the tumor size, the higher the LIF expression; however, no significant association was observed between LIF expression and tumor size (*p* = 0.051). We also found a significant association of LIF protein expression and other clinicopathological variables such as depth of invasion (*p* = 0.001) and surgical margins (*p* = 0.023). Furthermore, univariate analysis was used to investigate the relationships of LIF expression and cancer characteristics with patients’ overall survival (Figure 2). Kaplan–Meier curves showed that OSCC patients with higher LIF expression, advanced stage, large tumor size, or positive lymph node metastasis had significantly shorter overall survival (*p* < 0.001, *p* = 0.011, *p* = 0.002, and *p* = 0.014, respectively; log-rank test) than others. Univariate and multivariate survival analyses were performed using a Cox proportion hazards regression model. Advanced lymph node metastasis (*p* = 0.041), poor histological differentiation (*p* = 0.027), a DOI of 5–9 mm (*p* = 0.007), a DOI of <5 mm (*p* = 0.001), and advanced clinical stage (*p* = 0.001) were correlated with poor survival in the univariate analysis. Advanced clinical stage (*p* = 0.026) was identified as an independent unfavorable prognosis factor in the multivariate analysis (Table 2). In addition, the association between LIF and habits was evaluated. The details of patients’ oral health habits, including the daily or weekly consumption of areca quid (AQ), cigarettes, and alcohol, as well as the duration of these habits, were recorded. Patients with OSCC were defined as AQ chewers when they chewed two or more AQs daily for at least 1 year. They were categorized as cigarette smokers when they smoked every day for at least 1 year and consumed more than 50 packs of cigarettes per year. Finally, they were classified as alcohol drinkers when they drank more than 4 days a week and consumed more than 20 g of pure alcohol per week for at least 1 year. According to these definitions, 79 (79%) were drinkers, 80 (80%) were AQ chewers, and 83 (83%) were smokers. Finally, no significant correlation was found between LIF expression and areca nut chewing, tobacco smoking, and alcohol drinking (Table 3). This finding suggests that LIF plays an important role in the cancer progression of patients with OSCC.

### 3.2. LIF Enhances Cell Migration and Invasion Abilities through Outside-In Signaling in OSCC Cells

**Figure 3:**
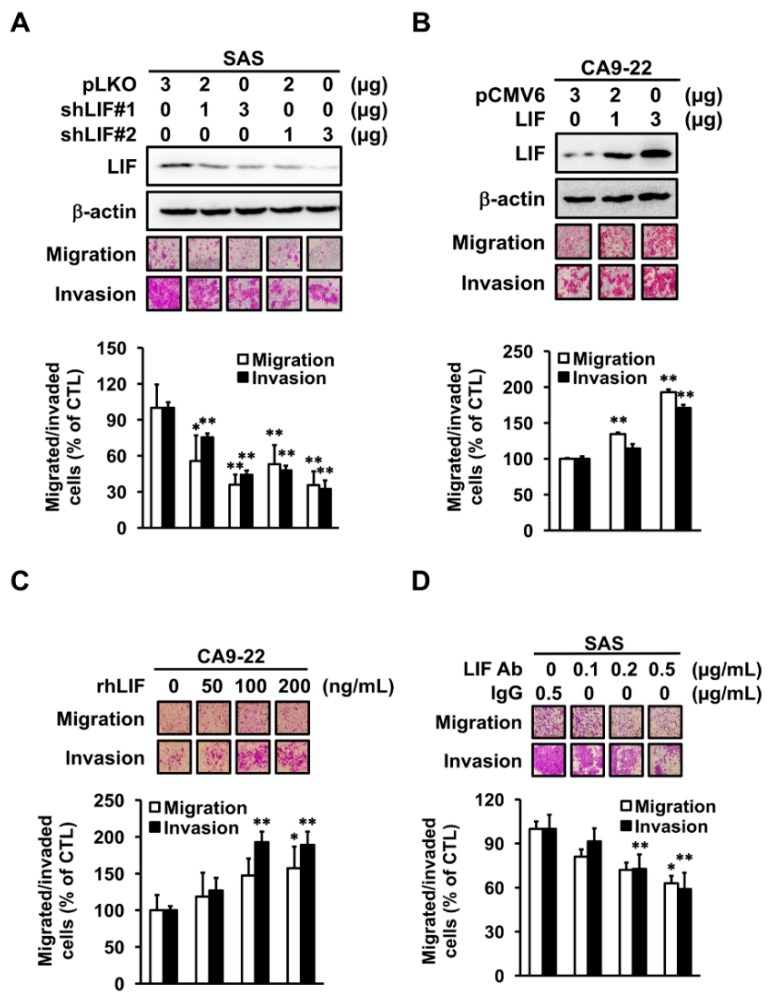
Invasion and migration ability of LIF in OSCC cells. (**A**,**B**) Western blot analysis of LIF expression in CA9-22 or SAS cells transiently transfected with LIF-expressed or shLIF plasmid. β-actin was used as an internal control. An in vitro migration and invasion assay was used to evaluate cell migration and invasion ability, performed for 24 or 48 h (* *p* <0.05, ** *p* < 0.01). (**C**,**D**) CA9-22 and SAS cells were subcultured in a Boyden chamber and treated with various concentrations of recombinant LIF protein (rLIF) or LIF neutralization antibody overnight. Cell motility toward the lower face of the filter was observed and quantified (* *p* < 0.05, ** *p* < 0.01).

To clarify the correlation between LIF expression and cancer metastasis in oral cancer, we used OSCC cell lines to investigate whether LIF plays a critical role in OSCC progression. We first knocked down LIF expression by shRNA. Transiently transfected shLIF expression plasmid in SAS cells indicated that migrated and invaded cells were decreased compared with control cells (Figure 3A). We also knocked down LIF expression in Cal27 cell lines and confirmed that the cell motility was decreased in another cell line (Figure S1). By contrast, the invasion and migration capabilities were enhanced in LIF overexpressing CA9-22 cells compared with control cells (Figure 3B).

LIF can be secreted and detected in the cell matrix [26]. To confirm if LIF regulates cell motility through outside-in or inside-out signaling, recombinant LIF protein (rLIF) and LIF neutralization antibody were used. rLIF significantly increased the migration and invasion of CA9-22 cells in a dose-dependent manner (Figure 3C). In addition, LIF neutralization antibody decreased the motility of SAS cells in a dose-dependent manner (Figure 3D), indicating that LIF induced cell motility through outside-in signaling. With such results taken together, we concluded that LIF has direct roles in enhancing OSCC metastasis.

### 3.3. INHBA is a Key Downstream Effector in LIF-Enhanced OSCC Progression

**Figure 4:**
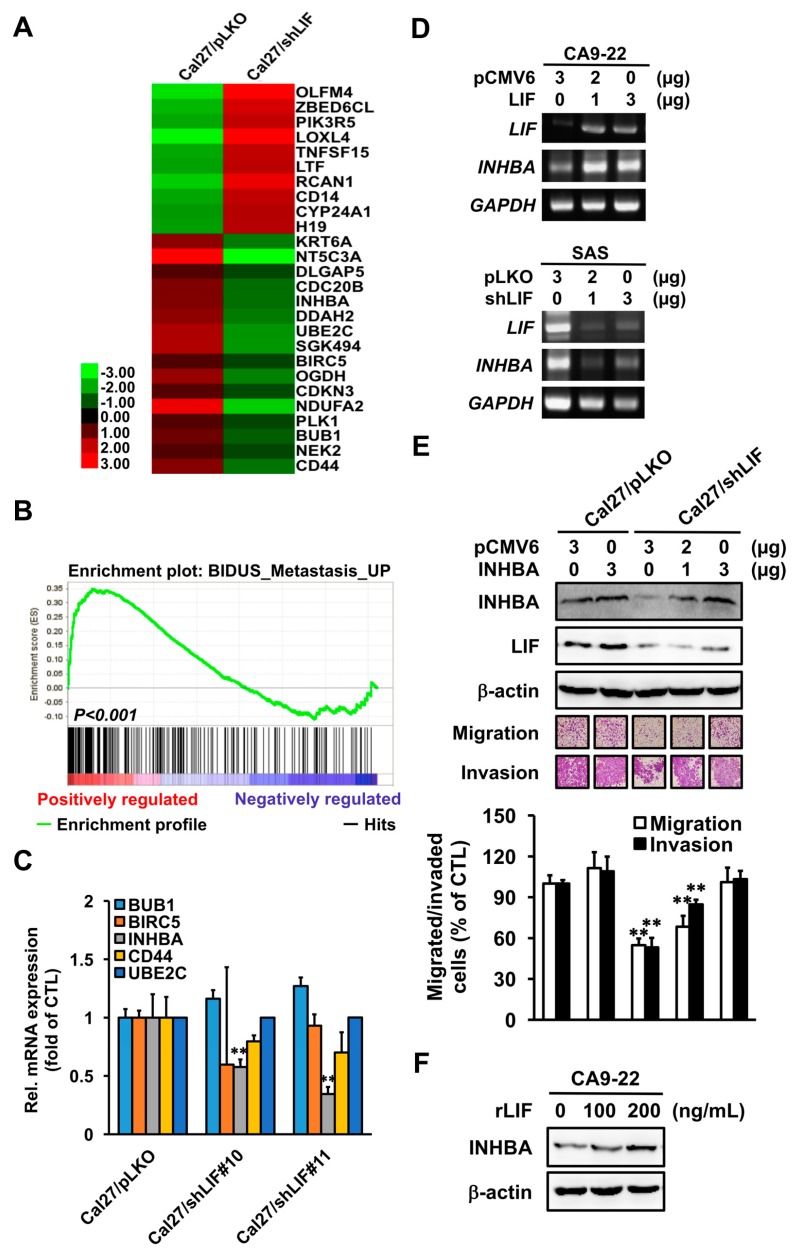
Inhibin beta A subunit (INHBA) as the major downstream effector in LIF increases oral cancer progression. (**A**) Heatmap of mRNA expression profile in Cal27/pLKO and Cal27/shLIF stable clones. (**B**) Gene set enrichment analysis (GSEA) showed the enrichment of metastatic genes in Cal27/pLKO versus Cal27/shLIF cells. (**C**) Real-time PCR analysis of *BUB1*, *BIRC5*, *INHBA*, *CD44*, and *UBE2C* mRNA expression in Cal27/pLKO and Cal27/shLIF cells (** *p* < 0.01). (**D**) Reverse transcription PCR analysis of *INHBA* mRNA expression in CA9-22 and HSC3 cells transiently transfected LIF-expressed or shLIF plasmids. (**E**) Cal27/pLKO and Cal27/shLIF stable clones were seeded and transiently transfected with 3 µg of control plasmid or various concentrations of INHBA plasmids and incubated for 48 h, then subcultured in a Boyden chamber overnight. Cell motility toward the lower face of the filter was observed and quantified (** *p* < 0.01). (**F**) Western blot analysis of INHBA protein expression in CA9-22 cells after they were treated with rLIF. β-actin was used as an internal control.

As LIF induced tumor cell metastasis ability, we investigated the potential downstream effector(s) of LIF in OSCC progression; thus, a mRNA microarray was performed. According to Figure 4A, the heatmap of stable LIF transfectants showed 22 genes that were substantially regulated by LIF. To further analyze the function of LIF in our array data, gene set enrichment analysis (GSEA) was conducted, which showed that the metastasis-associated genes were significantly downregulated in LIF knockdown Cal27 cells compared with control clone (Figure 4B). We next validated the mRNA expression of metastasis-related genes in the microarray analysis, including *ubiquitin-conjugating enzyme E2 C* (*UBE2C*), *INHBA*, *CD44 molecule* (*CD44*), *mitotic checkpoint serine/threonine kinase* (*BUB1*), and *baculoviral IAP repeat containing 5* (*BIRC5*) by quantitative real-time PCR (Figure 4C). The mRNA expression of *INHBA* was remarkably decreased in LIF knockdown Cal27 cells. As observed with microarray findings, the *INHBA* mRNA expression was upregulated by overexpressed LIF and downregulated by knockdown LIF expression in OSCC cell lines (Figure 4D). We thus hypothesized that *INHBA* might be a potential downstream target gene of LIF.

To evaluate whether INHBA is a major downstream effector in LIF-induced OSCC metastasis, we transiently transfected IHNBA-expressing plasmids with LIF knockdown transfectants in a dose-dependent manner and then determined the cells’ migration and invasion abilities. This transient transfection with INHBA plasmids in LIF knockdown transfectants substantially restored the shLIF-decreased INHBA protein expression and cell motility (Figure 4E). To investigate whether the INHBA protein expression was regulated by LIF through outside-in signaling, rLIF treatment of CA9-22 cells was performed, which considerably increased the INHBA protein expression (Figure 4F). These findings suggest that INHBA is a potential downstream effector in LIF-induced OSCC cancer progression.

### 3.4. Relationship between *LIF* and *INHBA* Expression in Patients with OSCC

**Figure 5:**
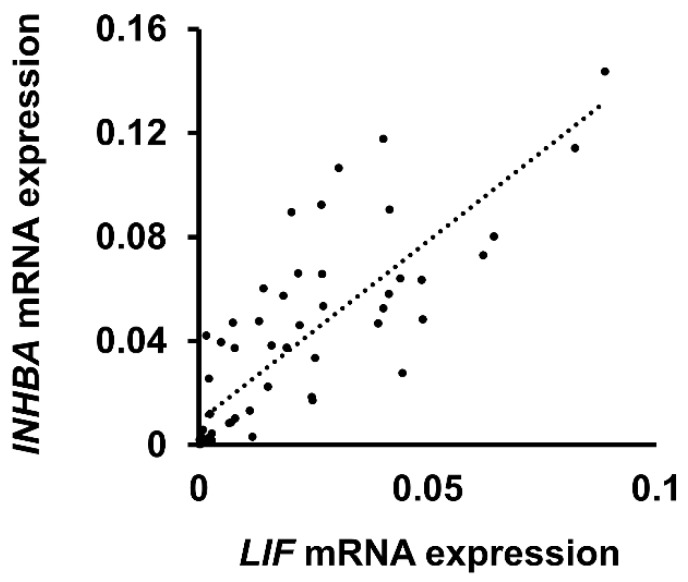
*INHBA* was moderately correlated with *LIF* in patients with OSCC. Correlation between *LIF* and *INHBA* in patients with OSCC (*R*^2^ = 0.67). The mRNA levels of patients’ samples were measured using real-time PCR. Data are presented as mean ± SD for mRNA expression.

To validate our findings, we determined the correlation between *LIF* and *INHBA* mRNA expression in patients with oral cancer by quantitative real-time PCR. As Figure 5 shows, *INHBA* mRNA expression was moderately correlated with *LIF* mRNA expression in patients with OSCC (*R*^2^ = 0.67). The result supports the assumption that, in vitro, INHBA influences LIF-associated metastasis in oral cancer.

## 4. Discussion

LIF has been well studied as a tumorigenesis promotor or suppressor in different types of tumors. OSCC is the most prevalent malignant tumor of the head and neck region. Despite improvements in treatment, the five-year survival rate of patients with oral cancer has still not improved significantly. A major challenge of current basic and clinical research is to identify a novel molecular marker that can improve the treatment of oral cancer.

In this study, we identified a relationship between LIF and major risk factors for oral cancer such as tobacco smoking, betel nut chewing, and alcohol consumption. The results demonstrated that no significant correlation existed between LIF and these habits. On the basis of our findings, LIF was not involved in the mechanisms of tobacco smoking, betel nut chewing, and alcohol consumption related to oral cancer. Whether LIF participates in carcinogenesis remains controversial. However, we found that LIF is essential for oral cancer transformation and malignant progression. LIF can promote cancer cell progression, including migration and invasion, through INHBA in OSCC cells. INHBA encoding inhibin β A subunit dimerizes with another β subunit to make activin. Activin enhances Follicle Stimulating Hormone (FSH) biosynthesis and secretion and participates in menstrual cycle regulation [27]. Studies have revealed that activin is overexpressed in OSCC compared with normal oral mucosa, and that high activin A levels are significantly associated with lymph node metastasis, tumor differentiation, and poor survival [28,29]. LIF overexpression enhanced INHBA expression to regulate cancer cell motility, whereas LIF knockdown reduced cell motility and INHBA expression in shLIF transfectants. This suggests that LIF and INHBA are essential for OSCC progression. We found that patients with positive lymph node metastasis had higher LIF expression. However, high LIF staining was also found in patients with advanced cancer stages. These findings suggest that LIF plays an important role in the cancer progression of patients with OSCC.

The role of LIF in multiple cancers is variable. It acts as an oncogene in rhabdomyosarcoma [24], nasopharyngeal carcinoma [18], and colorectal cancer [25], but it acts as a tumor suppressor in several cancer types, including breast cancer [22,23], melanoma [21], and hepatocellular carcinoma [30]. Research has indicated that some genes have both oncogenic and tumor-suppressing potential in various cancer types [31,32,33,34]. Cancer is a somatic mutation accumulation [35,36]. It has been mentioned that the gain-of-function mutations of these genes that hold dual roles might promote oncogenic functions, and the loss-of-function mutations downregulate the tumor-suppressing expression [31]. Additionally, the microenvironment around a tumor determines the direction of tumor development and metastasis [33]. The complex immune networks of immune cells and the cytokines surrounding the tumors influence the outcomes of anticancer activities or tumor promotion [33,37]. A gene can become a double-edged sword by either promoting or inhibiting tumor progression according to cancer types. Identifying the interaction between genes and cancers in the tumor microenvironment is crucial.

Ohata et al. demonstrated that OSCC stimulated cancer-associated fibroblasts (CAFs) to produce LIF, which induced cell invasion. However, the mechanism through which LIF promotes cancer motility remains unclear [38]. Based on our findings, rLIF and the transfection of LIF expression vector confirmed that both exogenous and endogenous LIF induced oral cancer motility. We identified *INHBA* as a potential downstream gene in LIF-associated OSCC progression using a high-throughput mRNA microarray. In a previous study, the overexpression of activin A in OSCC regulated cell apoptosis and invasiveness [28]. Activin A was also clinically correlated with lymph node metastasis and poor survival [28]. Therefore, we posit that CAF-produced or OSCC-produced LIF may be the upstream effector of INHBA in the control of OSCC metastasis (cell motility and invasion).

The results of the present study revealed that almost half of the patients with stage 4 cancer had low LIF expression (17/39). We observed that stage 4 patients with lower LIF expression had lower rates of regional lymph node metastasis and a longer survival time compared with stage 4 patients with high LIF expression. These findings also imply that patients who express low LIF tend to have a good prognosis. Although LIF expression does not exhibit a significant association with tumor status, higher LIF protein levels seem to be associated with more invasive cancer behavior, such as deeper invasion and regional lymph node metastasis. In clinical practice, if a patient presents with a small primary tumor with high LIF expression, the possibility of regional lymph node metastasis should be a concern. These findings suggest that LIF can be a valuable predictor in patients with local invasive cancer behavior, even in cases where the tumor status is not advanced.

## 5. Conclusions

In conclusion, LIF contributes to cancer progression by enhancing regional lymphatic spread, thereby leading to an advanced cancer stage. Regulation of LIF downstream molecules, such as INHBA, can thus inhibit cell invasion and migration. We demonstrated a novel mechanism (INHBA regulation) by which LIF influences cancer progression. LIF can thus be a potential diagnostic or therapeutic marker in patients with advanced OSCC stages.

## Figures and Tables

**Table 1:** Correlation between leukemia inhibitory factor (LIF) expression and clinicopathological parameters.

Clinicopathological Parameters	Degree of LIF Staining			
	0 (*n* = 32)	1 or 2 (*n* = 22)	3 or 4 (*n* = 46)	*p*-Value ^a^
Patient’s ages (years)				
<50	7 (21.9%)	6 (27.3%)	11 (23.9%)	0.828
50–59	18 (56.2%)	9 (40.9%)	19 (41.3%)	
60–69	4 (12.5%)	5 (22.7%)	9 (19.6%)	
≥70	3 (9.4%)	2 (9.1%)	7 (15.2%)	
Patient’s sex				
Male	28 (87.5%)	19 (95.5%)	44 (95.6%)	0.335
Female	4 (12.5%)	3 (4.5%)	2 (4.4%)	
Cancer locations				
Buccal and lip SCC	16 (50%)	10 (45.5%)	22 (47.8%)	0.526 ^b^
Gingival SCC	5 (15.6%)	5 (22.7%)	2 (4.3%)	
Mouth floor SCC	0 (0%)	0 (0%)	1 (2.2%)	
Palate SCC	1 (3.1%)	1 (4.5%)	3 (6.5%)	
Tongue SCC	10 (31.3%)	6 (27.3%)	18 (39.1%)	
T status				
T1–T2	22 (68.8%)	19 (86.4%)	23 (50%)	0.051
T3–T4	10 (31.2%)	3 (13.6%)	23 (50%)	
N status				
N0	23 (71.9%)	12 (54.5%)	18 (39.1%)	0.022 *
N1	6 (18.8%)	4 (18.2%)	20 (43.5%)	
N2–N3	3 (9.3%)	6 (27.3%)	8 (17.4%)	
Clinical staging				
Stage 1	10 (31.25%)	5 (22.7%)	5 (10.9%)	0.022 *
Stage 2	10 (31.25%)	7 (31.8%)	6 (13.0%)	
Stage 3	2 (6.25%)	3 (13.6%)	13 (28.3%)	
Stage 4	10 (31.25%)	7 (31.8%)	22 (47.8%)	
Stages 1–2	20 (62.5%)	12 (54.5%)	11 (23.9%)	0.002 *
Stages 3–4	12 (37.5%)	10 (45.5%)	35 (76.1%)	
Histological differentiation				
Well-diff. SCC	30 (93.8%)	20 (90.1%)	41 (89.1%)	0.773
Moderately-diff. SCC	1 (3.1%)	2 (9.9%)	3 (6.5%)	
Poorly-diff. SCC	1 (3.1%)	0 (0.0%)	2 (4.4%)	
Depth of invasion (DOI)				
<5 mm	25 (78.1%)	13 (59.1%)	9 (19.6%)	0.001 *
5–9 mm	5 (15.6%)	5 (22.7%)	29 (63.0%)	
>9 mm	2 (6.3%)	4 (18.2%)	8 (17.4%)	
Margin status				
≥5 mm	29 (90.6%)	17 (72.3%)	27 (58.7%)	0.023 *
<5 mm	1 (3.1%)	4 (18.2%)	15 (32.6%)	
Involved	2 (6.3%)	1 (4.5%)	4 (8.7%)	
Perineural invasion				
No	27 (84.4%)	18 (81.8%)	36 (78.3%)	0.79
Yes	5 (15.6%)	4 (18.2%)	10 (21.7%)	

^a^ Kruskal–Wallis test, ^b^ Based on a chi-squared test. Abbreviation: SCC—squamous cell carcinoma. (* *p* < 0.05).

**Table 2:** Univariate and multivariate survival analyses of LIF and clinicopathological parameters in patients with oral squamous cell carcinoma (OSCC).

Factor	Hazard Ratio (95% CI)	*p*-Value ^a^
Univariate		
Cancer locations (palatal vs. buccal and lip)	4.95 (0.92–26.88)	0.062
Cancer locations (palatal vs. gingival)	3.52 (0.46–26.73)	0.22
Cancer locations (palatal vs. tongue)	4.18 (0.53–32.68)	0.172
T status (T3 + T4 vs. T1 + T2)	1.44 (0.30–6.94)	0.217
N status (N2 + N3 vs. N0)	2.78 (0.37–20.45)	0.314
N status (N2 + N3 vs. N1)	2.91 (1.04–8.06)	0.041 *
Clinical staging (stages 3 + 4 vs. 1 + 2)	0.48 (0.06–3.64)	0.481
Histological differentiation (poor vs. well)	13.10 (1.35–127.41)	0.027 *
Histological differentiation (moderate vs. well)	1.17 (0.31–4.33)	0.81
DOI (>10 mm vs. 5–9 mm)	5.43 (1.58–18.66)	0.007 *
DOI (>10 mm vs. <5 mm)	16.08 (2.96–87.71)	0.001 *
Margin status (margin involved vs. A > 5 mm)	1.88 (0.38–9.26)	0.435
Margin status (margin involved vs. closed < 5 mm)	4.95 (0.71–34.48)	0.104
PNI (negative vs. positive)	0.87 (0.28–2.72)	0.817
LIF label index (3 + 4 vs. 0)	8.84 (5.71–136.99)	0.001 *
LIF label index (3 + 4 vs. 1 + 2)	1.36 (0.0–2.7)	0.05
Multivariate		
LIF Label index (3 + 4 vs. 0)	6.83 (2.88–133.68)	0.026 *

^a^ Based on a Cox regression—proportion hazards model test. Abbreviation: CI—confidence interval. PNI: Perineural Invasion. (* *p* < 0.05).

**Table 3:** Correlation between LIF expression and habits of betel nut chewing and tobacco smoking.

Clinicopathological Parameters	Degree of LIF Staining			
	0 (*n* = 32)	1 or 2 (*n* = 22)	3 or 4 (*n* = 46)	*p*-Value ^a^
Daily alcohol consumption				
Nondrinkers	6 (18.8%)	5 (22.7%)	10 (21.7%)	0.997
≤3500 mL	17 (53.1%)	11 (50%)	23 (50.00%)	
>3500 mL	9 (28.1%)	6 (27.3%)	13 (28.3%)	
Duration of drinking alcohol				
Nondrinkers	6 (18.8%)	5 (22.7%)	10 (21.7%)	0.794
≤10 years	7 (21.9%)	7 (31.8%)	9 (19.6%)	
>10 years	19 (59.3%)	10 (45.5%)	27 (58.7%)	
Daily AQ consumption				
Nonchewers	10 (31.3%)	4 (18.2%)	5 (10.9%)	0.176
≤10 quids	10 (31.2%)	7 (31.8%)	22 (47.8%)	
>10 quids	12 (37.5%)	11 (50%)	19 (41.3%)	
Duration of chewing AQs				
Nonchewers	10 (31.3%)	4 (18.2%)	5 (10.9%)	0.219
≤10 years	5 (15.6%)	6 (27.3%)	13 (28.3%)	
>10 years	17 (53.1%)	12 (54.5%)	28 (60.8%)	
Daily cigarette consumption				
Nonsmokers	6 (18.8%)	3 (13.6%)	8 (17.4%)	0.487
≤1 pack	17 (53.1%)	15 (68.2%)	21 (45.6%)	
>1 pack	9 (28.1%)	4 (18.2%)	17 (37.0%)	
Duration of smoking				
Nonsmokers	6 (18.8%)	3 (13.6%)	8 (17.4%)	0.092
≤10 years	2 (6.2%)	2 (9.1%)	4 (8.7%)	
>10 years	24 (75%)	17 (77.2%)	34 (73.9%)	

^a^ Based on a chi-square test. Abbreviation: AQ—areca quid.

## Supplementary Materials

The following are available online at https://www.mdpi.com/2077-0383/9/2/295/s1, Figure S1: Knocked down LIF expression decreased the invasion and migration capabilities in Cal27 cell lines.
