# Supplementary material for: Role and Mechanism of LIF in Oral Squamous Cell Carcinoma Progression
Source: J Clin Med. 2020 Jan 21;9(2):295. doi: 10.3390/jcm9020295 (PMC7073607; doi:10.3390/jcm9020295)

Figure S1. Knocked down LIF expression decreased the invasion and migration capabilities in Cal27 cell lines. (** $p < 0.01$).

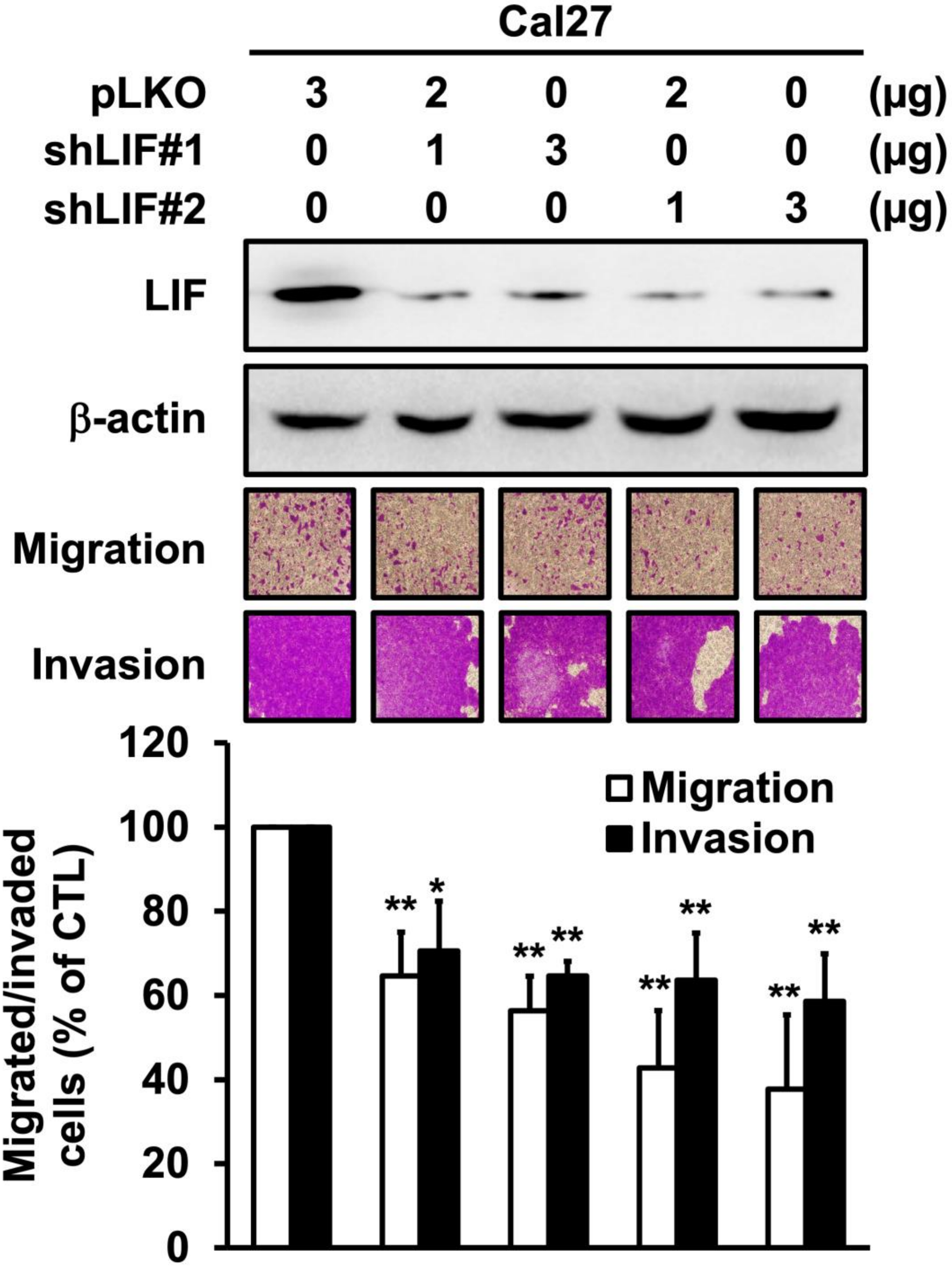

Supplement: Supplementary file 1 [file jcm-09-00295-s001.pdf]
